# Enhanced Chemical and Electrochemical Stability of Polyaniline-Based Layer-by-Layer Films

**DOI:** 10.3390/polym13172992

**Published:** 2021-09-03

**Authors:** Putri Bintang Dea Firda, Yoga Trianzar Malik, Jun Kyun Oh, Evan K. Wujcik, Ju-Won Jeon

**Affiliations:** 1 Department of Chemistry, Kookmin University, 77, Jeongneung-ro, Seongbuk-gu, Seoul 136-702, Korea; putribdf@kookmin.ac.kr (P.B.D.F.); yogamalik@kookmin.ac.kr (Y.T.M.); 2 Department of Polymer Science and Engineering, Dankook University, 152 Jukjeon-ro, Suji-gu, Yongin-si 16890, Korea; junkyunoh@dankook.ac.kr; 3 Materials Engineering and Nanosensor [MEAN] Laboratory, Department of Chemical and Biological Engineering, The University of Alabama, Tuscaloosa, AL 35487, USA; evan.wujcik@ua.edu

**Keywords:** polyaniline, polyacid, doping, layer-by-layer assembly, chemical stability, electrochemical stability

## Abstract

Polyaniline (PANI) has been widely used as an electroactive material in various applications including sensors, electrochromic devices, solar cells, electroluminescence, and electrochemical energy storage, owing to PANI’s unique redox properties. However, the chemical and electrochemical stability of PANI-based materials is not sufficiently high to maintain the performance of devices under many practical applications. Herein, we report a route to enhancing the chemical and electrochemical stability of PANI through layer-by-layer (LbL) assembly. PANI was assembled with different types of polyelectrolytes, and a comparative study between three different PANI-based layer-by-layer (LbL) films is presented here. Polyacids of different acidity and molecular structure, i.e., poly(acrylic acid) (PAA), polystyrene sulfonate (PSS), and tannic acid (TA), were used. The effect of polyacids’ acidity on film growth, conductivity, and chemical and electrochemical stability of PANI was investigated. The results showed that the film growth of the LbL system depended on the acidic strength of the polyacids. All LbL films exhibited improved chemical and electrochemical stability compared to PANI films. The doping level of PANI was strongly affected by the type of dopants, resulting in different chemical and electrochemical properties; the strongest polyacid (PSS) can provide the highest conductivity and chemical stability of conductive PANI. However, the electrochemical stability of PANI/PAA was found to be better than all the other films.

## 1. Introduction

Polyaniline (PANI) is a type of conducting polymer that has been widely studied in sensors, electrochromic devices, solar cells, electroluminescence, and electrochemical energy storage systems due to its tunable conductivity, redox properties, high chemical flexibility, low cost, and facile synthesis [1,2,3,4,5]. Through a unique doping–dedoping process, PANI can have different oxidation states, i.e., fully reduced leucoemeraldine base (LB), leucoemeraldine salt (LS), partially oxidized emeraldine salt (ES), emeraldine base (EB), fully oxidized pernigraniline base (PB), and pernigraniline salt (PS), resulting in substantial changes in electrical conductivity and electroactivity [6]. The conductive state of PANI (ES form) can easily lose its electrical conductivity and electroactivity by dedoping and/or excessive oxidation [7,8]. For example, the conductive ES form of PANI can be spontaneously converted to a nonconductive EB form due to the volatility of small molecule dopants [9,10]. In addition, PANI can lose its electroactivity by diffusion of dopants and irreversible oxidation, resulting in performance degradation [7,8,9]. The low chemical and electrochemical stability of PANI is disadvantageous in many cases, which hinders its more practical applications [11,12]. Therefore, it is essential to improve PANI’s chemical and electrochemical stability, which remains a challenge.

One of the ways to increase the chemical and electrochemical stability of PANI is to dope it using large molecule polyacids, instead of small molecule anion dopants [1,13,14,15]. The Loo Group reported that electroactivity and electrochemical stability can be enhanced by doping PANI using a strong polyacid, poly(2-acrylamide-2-methyl-1-propane sulfonic acid (PAAMPSA)), showing that PANI can reversibly change its oxidation state in neutral and alkaline aqueous solutions [9]. Other research reported that a sulfonic acid group of PAAMPSA can change its reaction pathway and improve electrochemical stability in an nonaqueous solution by preventing the irreversible formation of an excessively oxidized PB state [16].

Layer-by-layer (LbL) assembly is a technique that can produce multilayer films consisting of a wide range of materials including polyelectrolytes, nanoparticles, biomolecules, and colloids [17,18,19]. LbL assembly can provide versatile routes for the construction of functional materials on substrates by sequential deposition of electroactive materials [1,20,21]. Several electroactive PANI-based multilayer films have been reported through the LbL assembly technique: PANI/V_2_O_5_ [8], PANI/poly(acrylic acid) (PAA) [1], PANI/multiwalled carbon nanotube [20], and PANI/PAAMPSA [7] in which the enhancement of electrochemical stability was observed. For instance, by optimizing the LbL assembly condition, PANI doped using PAA, a weak polyacid with carboxylic acid functional groups, exhibited highly improved electrochemical stability, in a nonaqueous electrolyte, in comparison to PANI homopolymer [1]. These results indicate that secondary interactions between PANI and polyacids strongly affect the electrochemical properties of PANI. However, how polyacids’ characteristics affect the electrochemical properties of PANI-based film remains unclear. We hypothesize that characteristics of polyacids such as acidic strength and molecular structure can influence secondary interactions and the doping level of PANI, resulting in different chemical and electrochemical properties.

Herein, in order to improve chemical and electrochemical stability of PANI, we assemble three different PANI LbL films using polystyrene sulfonate (PSS), PAA, and tannic acid (TA). We perform a systematic study to investigate the effect of pKa and the structure of polyacids on the chemical and electrochemical properties of PANI. In this study, polyacids of different acidity and molecular structure, i.e., PSS, PAA, and TA, are used as dopants to fabricate PANI-based LbL films. PSS is a strong polyacid (pKa: 1) [21] and PAA is a weak polyacid (pKa: 4.5) [22], while TA is relatively small and the weakest polyacid (pKa: ~8.5) [23,24]. It is found that the acidic strength of polyacids assembled with PANI affects the doping level of PANI in a consistent manner. For PANI assembled using a strong polyacid, PSS successfully improved the chemical stability across a wide range of pH, while the highest electrochemical stability was achieved in PANI assembled with a weak polyacid, PAA.

## 2. Materials and Methods

### 2.1. Materials

Emeraldine base polyaniline (EB, Mw = 20,000), poly(acrylic acid) (PAA, Mw = 250,000, 35 wt.% in H_2_O), tannic acid (TA, Mw = 1701, ACS reagent), poly(ethyleneimine) (PEI, Mw = 750,000, 50% aqueous solution), dimethylacetamide (DMAc, anhydrous, 99.8%), sodium sulfate (Na_2_SO_4_, ACS reagent, anhydrous), and hydrochloric acid (HCl, ACS reagent, 37%) were purchased from Sigma-Aldrich (Seoul, Korea). Poly(styrene sulfonic acid) (PSS, Mw = 75,000, 30 *w*/*v*% aqueous solution) was purchased from Alfa Aesar (Tewksbury, MA, USA). Indium–tin–oxide (ITO)-coated glass (~25 Ω) was purchased from AMG glass.

### 2.2. Preparation of Dispersions

ES dispersion was prepared from EB according to the previously reported method [25]. Briefly, 0.05 g of EB was diluted to 10 mL of DMAc then stirred overnight and sonicated for 8 h. Afterwards, the dispersion was diluted using DI water at pH 3–3.5, at a ratio of 1:9 DMAc to DI water. Subsequently, the pH of the dispersion was adjusted to 2.5 using 1 M HCl. For 0.5 mg mL^−1^ solutions of PEI, PAA, TA, and PSS, 50 mg of materials was diluted separately using DI water until the volume reached 100 mL. Then the pH was adjusted to 2.5 using 1 M HCl. A stable PANI (ES) dispersion was obtained by controlling the pH of the prepared PANI (EB) dispersion at 2.5 using 1 M HCl. At a pH of 2.5, an EB state was converted to an ES state [16], which was confirmed by the observation of a color change from blue (EB) to green (ES) dispersion. The presence of an ES state was confirmed by the UV–Vis spectra that showed the characteristic peak of an ES spectra (Supplementary Materials Figure S1). The absorbance peak at ~840 nm indicates the electronic transition of C=N bond, a shoulder peak at ~420 nm is ascribed to the polaron band transitions, while an absorbance peak at ~340 nm is assigned to the π–π* transition of the benzenoid ring [26,27].

### 2.3. Glass Pretreatment

ITO-coated glass slides were sequentially washed with acetone, ethanol, and DI water. Nitrogen blowing was performed after each step to dry the glass surface. Subsequently, the glass was sonicated in DI water for 20 min to eliminate possible contaminants [28]. After being dried by nitrogen blowing, the glass was treated with UV-O_3_ treatment for 20 min to increase the hydrophilicity of the glass [29]. The cleaned glasses were then deposited with PEI–PAA bilayers. The glass was dipped into PEI solution for 5 min, and then dipped in DI water for 1 min, three times. Afterward, the PAA layer was deposited using the same method. This sequential process was repeated twice to deposit two bilayers of a PEI–PAA pair to improve the following film deposition. The presence of the carboxylic acid functional group of PAA can increase hydrophilicity of the glass surface, interacting with PANI so that the adhesion and stability of the LbL films are improved [20].

### 2.4. Preparation of PANI/Polyacid LbL Films and PANI Films

The as-prepared ITO-coated glass was dipped into PANI (ES) dispersion for 5 min, then, subsequently, immersed into DI water for 1 min; this was performed three times to remove the excess PANI that was adsorbed. The polyacid (PAA, TA, or PSSA) layer was deposited following the same procedure. A bilayer of positively charged PANI and negatively charged polyacids was deposited sequentially until the desired number of bilayers was reached. LbL films of 5, 10, 20, 30, and 40 bilayers were prepared for each sample. PANI homopolymer film was also prepared as a reference sample. PANI (ES) dispersion of 2.5 mg/mL was deposited to the as-prepared ITO-coated glasses using a spin-coating technique (2000 rpm, 30 s) until the desired thickness was obtained.

### 2.5. Characterization

Film thickness and roughness were measured using profilometry (D-500, KLA-Tencor). The measurement was carried out at six different points for each sample and the obtained value was then averaged. Fourier transform infrared (FTIR) spectroscopy was performed using benchtop FTIR spectrometer (Agilent Cary 630 FTIR) and samples were scanned using an ATR mode. UV–Vis spectra were measured using a PerkinElmer UV–Vis Lambda 356 spectrometer. The absorbance was recorded in the wavelength range of 300–1100 nm. The surface morphology of the LbL system was captured by field emission scanning electron microscopy (FE-SEM) using a JEOL JSM-7610F.

The electrochemical properties of the LbL films were investigated in an aqueous three-electrode cell. The LbL-coated ITO-coated glass was used as a working electrode, Ag/AgCl was used as a reference electrode, a platinum wire was used as the counter electrode, and 1 M Na_2_SO_4_ in water was used as the electrolyte. The electrochemical measurements were performed using an IVIUMnSTAT multichannel electrochemical analyzer. A cyclic voltammetry scan was carried out using a voltage range of −0.2 V to 0.8 V (vs. Ag/AgCl). Conductivity was measured by electrochemical impedance spectroscopy (EIS) using an IVIUMnSTAT multichannel electrochemical analyzer.

## 3. Results and Discussions

### 3.1. Physical Properties of LbL Films

**Figure 1:**
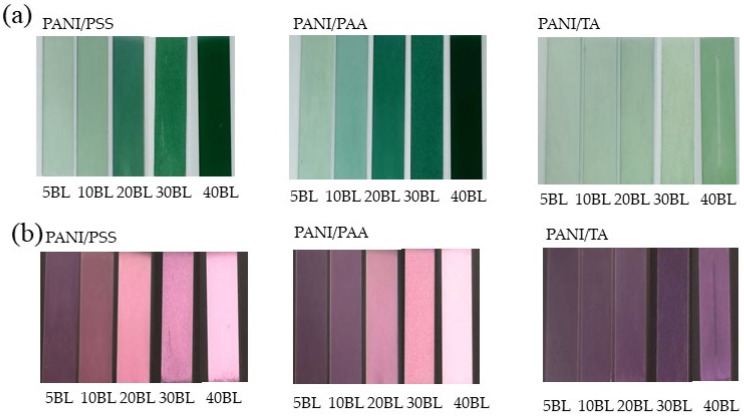

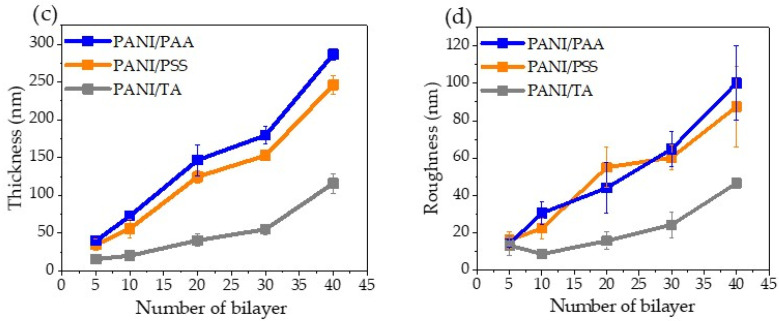
Photographs of the polyacid-doped PANI-based film: (**a**,**b**) the negative images, (**c**) growth profiles, and (**d**) roughness profiles of the LbL films of different bilayers (BL).

The photograph of the polyacid-doped, PANI-based film shows that, as the number of bilayers increases, a higher intensity of the film color (Figure 1a,b) is obtained, which indicates a consistent increase in film thickness. The growth and roughness profile are displayed by plotting film thickness and roughness as a function of bilayer numbers (Figure 1c,d). According to the profilometry results, the sample used for further characterizations is PANI/PAA 20BL, PANI/PSS 30BL, and PANI/TA 40BL which have similar thicknesses (116–150 nm).

Figure 1c shows the growth profiles of LbL films, in which PANI/PSS and PANI/PAA exhibit a similar growth pattern while PANI/TA film’s growth rate is much slower than PANI/PSS and PANI/PAA. The average thickness of one bilayer for PANI/PAA, PANI/PSS, and PANI/TA is 7, 5.8, 2.6 nm, respectively. The slow growth rate of PANI/TA can be attributed to the substantially smaller molecular weight of TA (Mw = 1701) compared to the other polyelectrolytes: PSS (Mw = 75,000) and PAA (Mw = 250,000). The slow growth rate of PANI/TA LbL films is confirmed by its weak green color (Figure 1a). The roughness value was obtained from the root-mean-square (RMS) data from profilometry measurement [30]. As shown in Figure 1d, the roughness profile of the LbL system also shows a similar trend to their growth profile.

**Figure 2:**
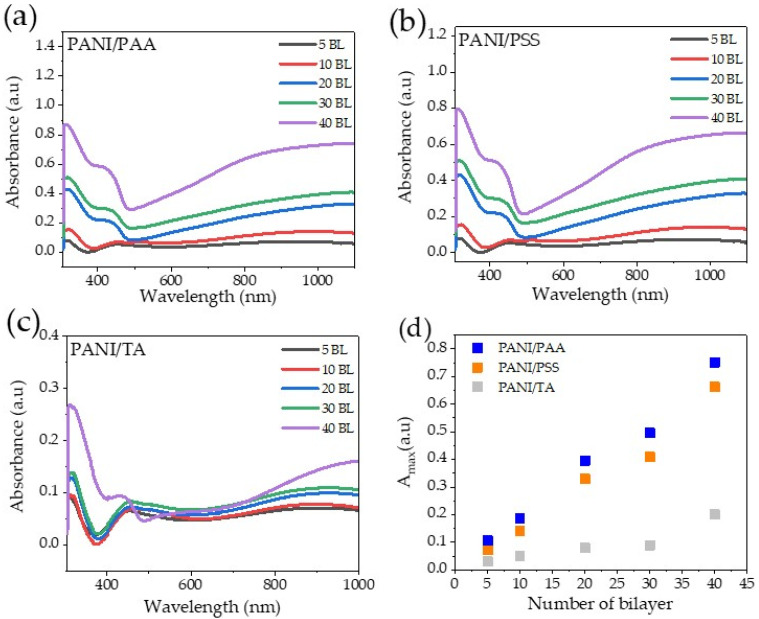
UV–Vis spectra of: (**a**) PANI/PAA, (**b**) PANI/PSS, and (**c**) PANI/TA LbL films and (**d**) their plot of maximum absorbance (~840 nm) as a function of the number of bilayers.

The UV–Vis spectra of the samples were recorded as a function of bilayer pairs (Figure 2a–c). The spectra of the LbL systems showed a similar characteristic to the UV–V is spectra of the ES solution (Figure S1), which indicates that the LbL assembly processes did not change the oxidation state of PANI (ES). The broad absorption band at ~840 nm confirmed the presence of ES. The trend of the absorbance intensity at ~840 nm increased as the number of bilayers increased (Figure 2d), which agrees positively with the growth profiles and the intensity of the film color (Figure 1a–c). According to the Beer–Lambert law, concentration of the light-absorbing species has proportional relationships based on the following equation:

(1) $$A=-\log10\frac{I}{I_{0}}=\mathrm{abC}$$

where A is absorbance, I is transmitted intensity, I_0_ is incident intensity, a is absorptivity (extinction coefficient), b is the length of the beam in the absorbing medium, and C is the concentration of the absorbing species [31,32]. This, therefore, reveals that the total amount of PANI consistently increases with an increased number of PANI/polyacid layer pairs.

**Figure 3:**
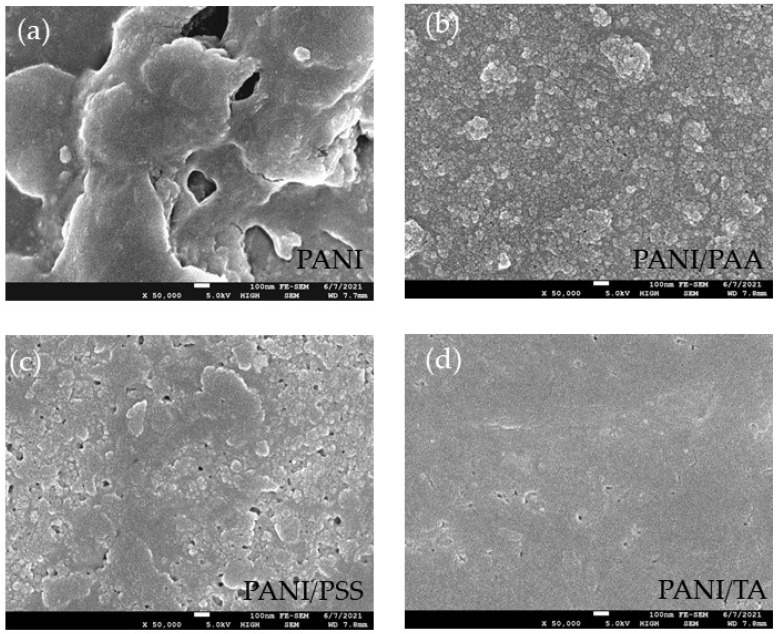
FE–SEM images of: (**a**) PANI, (**b**) PANI/PAA, (**c**) PANI/PSS, and (**d**) PANI/TA films. At a scheme of 100 nm.

The FE–SEM images of the LbL films and PANI film are displayed in Figure 3a–d, with a magnification of 50,000 times (scale bar of 100 nm). The PANI film showed large particle aggregation, while other LbL films showed relatively uniform surface morphology. The RMS roughness of PANI, PANI/PSS, PANI/PAA, and PANI/TA, with similar thickness (116–153 nm), is 125, 60, 47, and 44 nm, respectively. Previous studies showed that doping PANI using small molecule HCl renders high-electrical conductivity but undesirable aggregation, while weak organic acid doping leads to more uniform morphology but lower electrical conductivity [33]. 

### 3.2. Chemical Properties of PANI/Polyacids LbL Films and PANI Films

**Figure 4:**
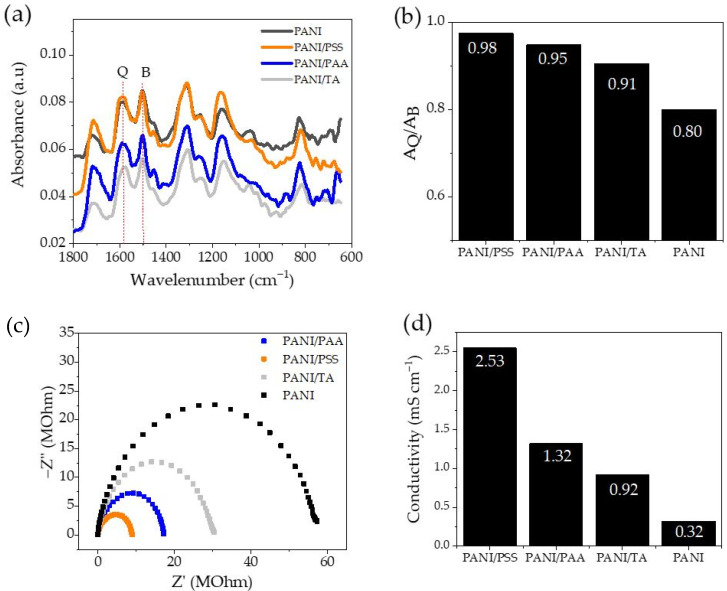
(**a**) FTIR spectra, (**b**) the ratio of quinoid to benzenoid peaks, (**c**) Nyquist plot, (**d**) conductivity value of doped PANI films.

The FTIR spectra of the LbL films and PANI film were collected using the ATR method using bare ITO for the baseline (Figure 4a). All recorded spectra included the characteristic peaks of PANI. As summarized in Table 1, the peaks at ~1590 cm^−1^ and ~1500 cm^−1^ are attributed to the -C=C- stretching of the quinoid (Q) ring and benzenoid (B) ring, respectively [34]. The peaks ~1310 cm^−1^ and ~1160 cm^−1^ are ascribed to the C-N vibration of secondary aromatic amine and -NH^+^ vibration from the protonation of nitrogen atoms in the imine ring of quinone, while the peak at ~820 cm^−1^ is attributed to the trans =C-H out-of-plane bending [35,36].

The doping level of PANI film can be investigated from the FTIR spectra by calculating the ratio of peak intensity of Q to B (Figure 4b). In the chemical structure of PANI, the B ring is the more reduced unit, while the Q ring is the more oxidized unit [37]. Thus, as the doping level of PANI increases, the ratio of Q to B increases. Therefore, the value of the peak intensity ratio (Q/B) can be used to examine the degree of doping of PANI [38,39]. All LbL films have a higher Q/B value than PANI homopolymer (Figure 4b). The PANI assembled using the strongest polyacid, PSS, exhibited a Q/B ratio of 0.98, which is substantially higher than that of PANI (0.80). This is consistent with the previous study showing that PANI doped with PAA and HCl have higher doping levels than PANI doped with HCl [40]. In our study, polyacid solutions also contained HCl; these were used to adjust the pH of the solutions. This possibly led to a co-doping effect of polyacids and HCl, resulting in an increased doping level.

Interestingly, in our study, the doping level of LbL films is consistent with the trend of pKa values of polyacids. The Q/B ratios of films are ordered: PANI/PSS (0.98) > PANI/PAA (0.95) > PANI/TA (0.91) > PANI (0.80). A higher doping level was obtained using a stronger polyacid, demonstrating lower pKa values. The pKa values of PSS, PAA, and TA are known to be ~1, ~4.5, and ~8.5, respectively; PSS is the strongest polyacid and TA is the weakest polyacid [21,22,23]. The proportional relationship between the doping level of PANI and the strength of the polyacids can be attributed to the fact that stronger polyacids have a greater amount of negatively charged functional groups, which can dope PANI more effectively.

EIS measurement was carried out at a frequency range of 1 Hz–250 kHz. The Nyquist plot is depicted in Figure 4c. The resistance (Rs) value was determined from the low-frequency intersection of the Nyquist plot with the Z’ axis. Then, the conductivity value was calculated using the following equation:

(2) $$\sigma =\frac{d}{RA}$$

where σ is conductivity (mS cm^−1^), *d* is electrode distance (cm), *R* is resistance (Ohm), and *A* is cross-sectional area (cm^−2^) [41]. The tabulated details of the data analysis are summarized in Table S2. As depicted in Figure 4d, the highest conductivity value of 2.53 mS cm^−1^ was obtained by PANI/PSS, followed by PANI/PAA at 1.32 mS cm^−1^, PANI/TA at 0.92 mS cm^−1^, and PANI at 0.32 mS cm^−1^. The trend of the conductivity value is consistent with the trend of quinoid to benzenoid peak ratio in the FTIR results in Figure 4b. The conductivity of PANI can vary depending on numerous variables such as synthesis method, doping level, etc. [42]. Previous research reported a conductivity value of PANI–PSS thin film in the range of 3 × 10^−4^ mS cm^−1^ [43]. In our case, the low conductivity value can be attributed to low film thickness, since the conductivity of the thin film decreases with decreasing film thickness [44]. Izet et al. reported that a reciprocal relationship between the rotational speed of the disk (rpm) and the specific conductivity of doped PANI film is inversely proportional; as the rpm increased (film thickness decreased), the conductivity of the film decreased [45].

The high conductivity of PANI makes it useful for various applications such as sensors, solar cells, thermoelectric, supercapacitors, and batteries [3,6,33,39]. Thus, it is important to improve the chemical stability of the conductive form of PANI (ES). It is known that the electroactivity of PANI will gradually reduce due to its oxidation state change from ES to EB by spontaneous dedoping of PANI [9]. The chemical stability of PANI depends on several factors such as acidity, molecular weight, polarity of dopants, pH condition, and type of electrolyte [46].

**Figure 5:**
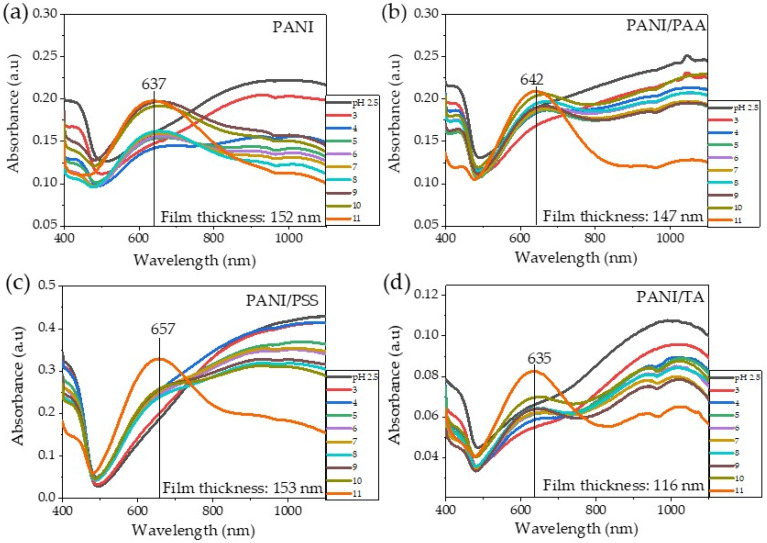
(**a**) UV–Vis spectra of PANI homopolymer, (**b**) PANI/PAA, (**c**) PANI/PSS, (**d**) and PANI/TA LbL films at different levels of pH in water.

We investigated the chemical stability of the ES state of PANI-based films using UV–Vis spectroscopy at different pH values of water, as shown in Figure 5a–d. The UV–Vis spectra of the PANI homopolymer film showed that peak intensity of a broad absorption peak at around 1000 nm significantly decreased, and a new peak at 637 nm started to appear at pH 5 (Figure 5a). This result indicates that the ES form of PANI films was converted to EB at a pH value higher than 5 [9]. Importantly, all PANI/polyacid LbL films exhibited better chemical stability of ES of PANI than the PANI homopolymer film. The PANI/PSS, PANI/PAA, and PANI/TA films can still retain the ES characteristic peak in the near-IR region (~1000 nm) up to pH 10. However, at pH 11, all LbL films exhibited substantially reduced peak intensity in the near-IR region, showing new peaks at 642, 635, and 657 nm, for PANI/PAA, PANI/TA, and PANI/PSS, respectively. The peaks at 642 nm (PANI/PAA) and 635 nm (PANI/TA) are indicative of EB [27], while the peak at 657 nm could be attributed to PS [16]. We hypothesize that the protonated amine group of PANI ES strongly interacts with the sulfonic acid group in PSS, more so than carboxylic acid in PAA and hydroxyl group in TA, which likely changes the reaction pathway.

### 3.3. Electrochemical Study of LbL Films

**Figure 6:**
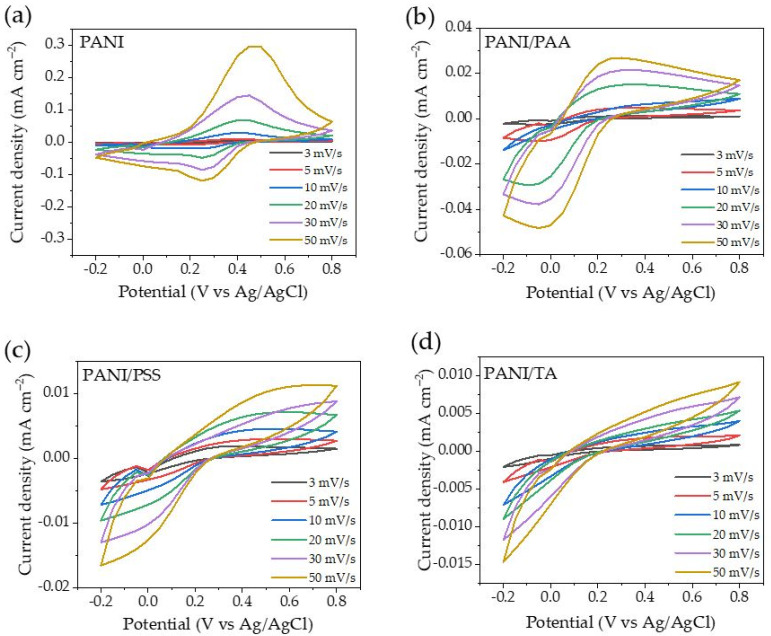
(**a**) Cyclic voltammograms of PANI, (**b**) PANI/PAA, (**c**) PANI/PSS, and (**d**) PANI/TA LbL films at different scan rates in 1 M Na_2_SO_4_ electrolyte (vs. Ag/AgCl reference electrode).

A cyclic voltammetry (CV) scan was conducted at different scan rates of 3, 5, 10, 20, 30, and 50 mV s^−1^ (Figure 6a–d) to investigate the nature of electrochemical reactions of the LbL films. Preconditioning was conducted by cycling at 10 mV s^−1^ five times until the cyclic voltammogram showed a stable current peak (data are not shown). The plot of the maximum current value as a function of scan rate and the square root of the scan rate was used to determine whether the electrochemical process was a diffusion-controlled process or not. The linear relationship between the current peak and the square root of the scan rate can be described by the Randles–Sevcik equation:

(3) $$I_{p}^{\mathrm{rev}}=\pm \;\left(2.69\times 10^{5}\right)n^{3/2}{\;\mathrm{ACD}}^{1/2}v^{1/2}$$

where $I_{p}^{\mathrm{rev}}$ is the current peak, n is the total number of electrons reacted per mole in the electrochemical process, A is film area (cm^2^), C is the concentration of the film material at the surface, D is the diffusion coefficient, and v is the scan rate [47]. While the linear relationship of the current peak and the scan rate can be characterized by the following equation:

(4) $$I_{p}=\;\frac{n^{2}F^{2}\mathrm{vlAC}}{2\mathrm{RT}}$$

where I_p_ is the current peak, n is the total number of electrons reacted per mole in the electrochemical process, F is faradaic constant, v is the scan rate, l is the distance of the film, A is the film area, C is concentration, R is the ideal gas constant, and T is temperature [47].

From the CV data, the maximum current is plotted vs. the square root of the scan rate and vs. the scan rate (Figure S2a–h). The linear relationship was determined by the value of R^2^ through data fitting. An R^2^ value closer to 1 means a stronger linear relationship between current peak and the square root of scan rates, or scan rates. A PANI sample shows a stronger linear relationship between current peak and scan rates, which suggests that a redox reaction of PANI films is not controlled by the diffusion of electrolytes; rather, it is controlled by its intrinsic redox properties [48,49]. In contrast, a stronger linear relationship between the maximum current peak and the square root of the scan rates was shown for PANI/PAA, PANI/PSS, and PANI/TA films, which suggests that the polyacid layers act as a physical barrier that hinders the diffusion of electrolytes to PANI [47,48].

**Figure 7:**
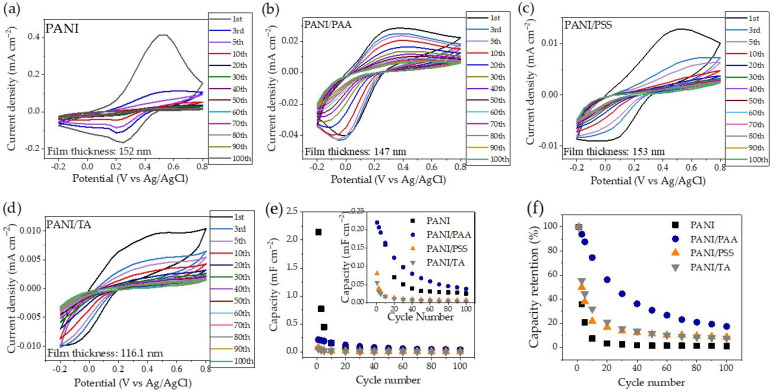
The cyclic voltammograms of (**a**) PANI, (**b**) PANI/PAA, (**c**) PANI/PSS, and (**d**) PANI/TA at the scan rate of 50 mV/s, and (**e**) a plot of areal capacitance, and (**f**) capacitance retention vs. cycle numbers.

Electrochemical activity and cycling stability were investigated by monitoring the areal capacitance value from the CV scans. In total, 100 cycles at a voltage range of −0.2 V to 0.8 V were carried out at a scan rate of 50 mV s^−1^. The result is displayed in Figure 7a–d. The areal capacitance value was calculated based on the following equation:

(5) $$\mathrm{Cs}=\frac{S}{2\left(\Delta V\right)\cdot A\cdot k}$$

where Cs, S, ΔV, A, and k are the areal capacitance, area of the CV loop, potential window (ΔV), film area (cm^2^), and scan rates (mV s^−1^), respectively [6,50]. Capacitance retention was calculated based on the following equation:

(6) $$\%\;\mathrm{capacitance}=\frac{C_{n}}{C_{0}}\times 100\%$$

where C_*n*_ is capacitance at n cycle and C_0_ is initial capacitance.

The plot of calculated capacitance and capacitance retention vs. cycle numbers is depicted in Figure 7e,f, while the details of the data analysis are summarized in Table S3. The results indicate that PANI had the highest initial capacitance of 2.145 mF cm^−2^, which significantly decreased to 0.769 mF cm^−2^ at the third cycle. The capacitance retention of the PANI sample was only 2% after 30 cycles. Conversely, all LbL films showed much lower initial capacitance values, but greatly improved cycling stability. The PANI/PSS had an initial capacitance of 8 × 10^−2^ mF cm^−2^, and it decreased by half to 4 × 10^−2^ mF cm^−2^ at the third cycle, while PANI/TA had the initial capacitance of 5.6 × 10^−2^ mF cm^−2^ and decreased to 2.6 × 10^−2^ mF cm^−2^ at the third cycle. The lower capacitance of PANI/PSS and PANI/TA films can be attributed to a lower PANI content compared to PANI homopolymer films. The capacitance retention of PANI/PSS and PANI/TA at the 30th cycle is 14% and 16%, respectively. In contrast, PANI/PAA has the highest capacitance retention of 45% at the 30th cycle, with an initial capacitance of 0.22 mF cm^−2^. This result is consistent with previous work that showed PANI/PAA improved electrochemical stability in a nonaqueous electrolyte [1]. The interactions between the carboxylic acid groups of PAA and the amine groups of PANI may mitigate the electrochemical degradation of films during cycling [1,51].

## 4. Conclusions

Three different PANI/polyacid LbL films were successfully fabricated by dip-assisted LbL assembly. The effect of the polyacids’ acidity on chemical and electrochemical properties of the LbL films was investigated and compared. It was found that pKa of polyacids are closely related to the doping level of PANI, showing that stronger acids can achieve a higher doping level of PANI. The LbL assembly of PANI using the strongest polyacid, PSS, provided the highest conductivity of 2.53 × 10^−3^ S cm^−1^ and improved the chemical stability of the doped ES form of PANI. In contrast, PANI/PAA outperformed PANI/PSS and PANI/TA in terms of electrochemical properties and electrochemical stability in an aqueous electrolyte. The PANI/PAA had the highest capacitance retention of 45% at the 30th cycle, which was an improvement on the PANI homopolymer under the same conditions. Our results suggest that the chemical and electrochemical stability of PANI can be improved using polyacid dopants, and the acidity of functional groups of polyacids affects the chemical (doping level and electrical conductivity) and electrochemical properties (capacitance and cycling stability) of PANI.

## Figures and Tables

**Table 1:** The associated peaks of the FTIR spectra from Figure 4 and the conductivity data.

Wavenumber (cm^−1^)				Characteristic
PANI	PANI/PSS	PANI/PAA	PANI/TA	
1592	1588	1592	1577	Quinoid ring stretching
1502	1506	1502	1502	Benzenoid ring stretching
1312	1312	1308	1308	C-N vibration of secondary aromatic amine
1163	1167	1159	1152	−NH^+^ vibration (protonation of nitrogen atoms in imine ring of quinones)
828	824	824	816	Trans = C-H out-of-plane bending

## Data Availability

All data in the manuscript and Supplementary Materials are available.

## Supplementary Materials

The following are available online at https://www.mdpi.com/article/10.3390/polym13172992/s1, Figure S1: UV-Vis spectra of emeraldine salt PANI. Figure S2: Plot of maximum current peak vs square root of scan rate, and maximum current peak vs scan rate of PANI (a,b), PANI/PAA (c,d), PANI/PSS (e,f), PANI/TA (g,h) from Figure 7. Table S1: Thickness data and roughness data of PANI/PAA (a,b), PANI/PSS (c,d), PANI/TA (e,f). Table S2: Data for conductivity calculation in Figure 4 (c,d), Table S3. Data of areal capacitance from Figure 7, with scan rate of 50 mV s^−1^ and voltage window of 1 V.
