# Supplementary material for: Enhanced Chemical and Electrochemical Stability of Polyaniline-Based Layer-by-Layer Films
Source: Polymers (Basel). 2021 Sep 3;13(17):2992. doi: 10.3390/polym13172992 (PMC8433781; doi:10.3390/polym13172992)
Supplement: Supplementary file 1 [file polymers-13-02992-s001.zip › polymers-1355825-supplementary.pdf]

Supporting Information

Enhanced Chemical and Electrochemical Stability of Polyaniline-Based Layer-by-Layer Films

Putri Bintang Dea Firda [1], Yoga Trianzar Malik [1], Jun Kyun Oh [2], Evan K. Wujcik [3], and Ju-Won Jeon [1*,]

[1] Department of Chemistry, Kookmin University, 77, Jeongneung-ro, Seongbuk-gu, Seoul 136-702, Republic of Korea

[2] Department of Polymer Science and Engineering, Dankook University, 152 Jukjeon-ro, Suji-gu, Yongin-si
16890, Gyeonggi-do, Republic of Korea

[3] Materials Engineering and Nanosensor [MEAN] Laboratory, Department of Chemical and Biological Engineering, The University of Alabama, Tuscaloosa, AL 35487, United States

* Correspondence: jwjeon@kookmin.ac.kr

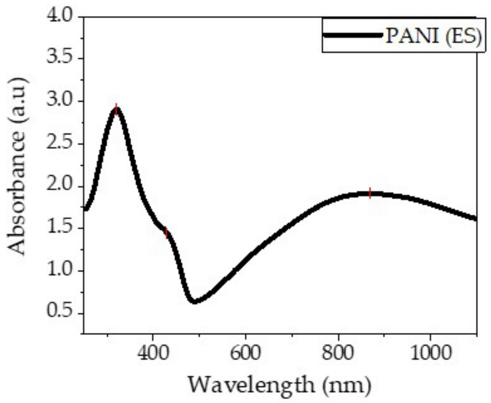

Figure S1. UV-vis spectra of emeraldine salt PANI

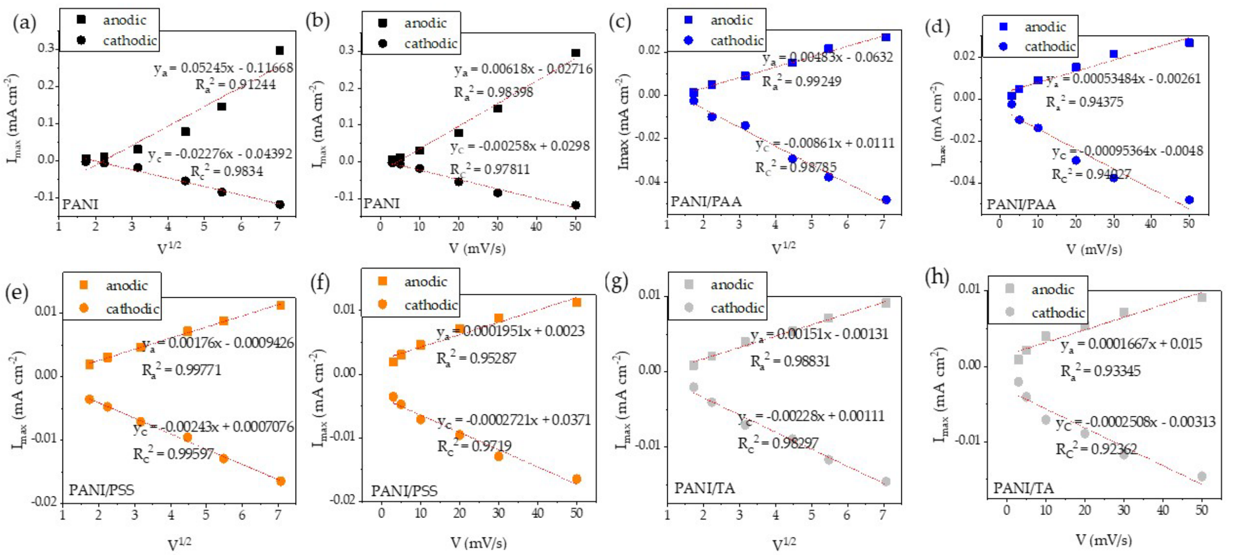

Figure S2. Plot of maximum current peak vs square root of scan rate,and maximum current peak vs scan rate of PANI (a, b); PANI/PAA (c, d); PANI/PSS (e, f), and PANI/TA (g, h) from Figure 7. a stands for anodic, c stands for cathodic.

Table S1a. Thickness data of the PANI/PAA sample

	thickness (nm)				
Point	5BL	10BL	20BL	30BL	40BL
1	38.3	83.8	134.76	174.47	280.58
2	45.7	75	164.9	186.27	282.73
3	32.85	65.8	179.5	184.54	279.39
4	37.05	72.9	138.44	158.32	299.41
5	43.75	68.3	132.6	187.57	287.87
6	41.9	70.7	130.23	185.82	291.9
Average	39.925	72.8	146.74	179.5	286.98
StdDev	4.750658	6.3166	20.435	11.403	7.696

Table S1b. Roughness data of the PANI/PAA sample

	roughness (nm)				
Point	5BL	10BL	20BL	30BL	40BL
1	15.5	38	34.345	61.756	78.759
2	13.9	36.6	68.339	76.27	125.62
3	11	22.4	51.361	72.968	100.81
4	14	26.9	39.661	56.545	110.9
5	16.7	29.6	34.002	52.06	110.26
6	14.7	30	37.122	69.661	74.385
Average	14.3	30.6	44.138	64.877	100.122
StdDev	1.923538	5.883	13.459	9.608	19.942

Table S1c. Thickness data of the PANI/PSS sample

	thickness (nm)				
Parameter	5BL	10BL	20BL	30BL	40BL
1	26.7	38.839	129.06	154.63	234.07
2	30.6	66.003	127.44	151.63	238.67
3	46.3	43.05	112.73	149.84	250.2
4	42.3	59.573	131.57	156.77	236.25
5	31.2	59.526	115.78	158.49	251.92
6	31.4	69.545	131.39	145.77	267.27
Average	34.8	56.089	124.66	152.855	246.3967
StdDev	7.697467	12.417	8.262	4.712094	12.61167

Table S1d.Roughness data of the PANI/PSS sample

	roughness (nm)				
Parameter	5BL	10BL	20BL	30BL	40BL
1	13.4	14.844	59.277	71.893	66.704
2	14.6	27.488	66.306	60.917	78.846
3	22.9	19.371	65.262	58.152	82.21
4	21.2	20.072	55.105	62.464	127.53
5	13.4	22.436	43.993	54.417	76.926
6	12.4	30.406	40.258	54.439	92.706
Average	16.3	22.436	55.034	60.38033	87.487
StdDev	4.5274	5.686	10.862	6.526828	21.33852

Table S1e. Thickness data of the PANI/TA sample

	thickness (nm)				
Parameter	5BL	10BL	20BL	30BL	40BL
1	12.5	20.6	35.3	55	130.4
2	17.4	24.5	28.2	51.2	105.9
3	11.3	16	50.5	52.2	99.2
4	17.9	21.1	39.5	48.4	108.7
5	16.1	24.2	44.2	67.8	124.6
6	18.5	14.4	46	56.6	127.7
Average	15.6	20.1	40.6	55.2	116.1
StdDev	3.0096	4.1645	8.0398	6.8118	13.0821

Table S1f. Roughness data of PANI/TA sample

	thickness (nm)				
Point	5BL	10BL	20BL	30BL	40BL
1	12.5	20.6	35.3	55	130.4
2	17.4	24.5	28.2	51.2	105.9
3	11.3	16	50.5	52.2	99.2
4	17.9	21.1	39.5	48.4	108.7
5	16.1	24.2	44.2	67.8	124.6
6	18.5	14.4	46	56.6	127.7
**Average**	**15.6**	**20.1**	**40.6**	**55.2**	**116.1**
StdDev	3.0096	4.1645	8.0398	6.8118	13.0821

Table S2. Data for conductivity calculation in Figure 4 (c, d)

	PANI/PSS	PANI/PAA	PANI/TA	PANI
thickness (cm)	1.53E-05	1.47E-05	1.16E-05	1.52E-05
electrode length (cm)	1.643	1.556	1.797	1.783
electrode distance (cm)	0.569	0.521	0.58	0.502
R (Ohm)	8.86E+06	1.73E+07	3.03E+07	5.73E+07
Conductivity (mS cm^{-1})	2.55	1.32	0.92	0.323

Table S3. Data of areal capacitance from Figure 7, with scan rate of 50 mV s^{-1} and voltage window of 1 V.

	PANI		PANI/PAA		PANI/PSS		PANI/TA	
cycle number	Curve area (mA.V cm^{-2})	Areal capacitance (mF cm^{-2})	Curve area (mA.V cm^{-2})	Areal capacitance (mF cm^{-2})	Curve area (mA.V cm^{-2})	Areal capacitance (mF cm^{-2})	Curve area (mA.V cm^{-2})	Areal capacitance (mF cm^{-2})
1	2.15E-01	2.15E+00	2.20E-02	2.20E-01	8.03E-03	8.03E-02	5.59E-03	5.59E-02
3	7.69E-02	7.69E-01	2.07E-02	2.07E-01	4.02E-03	4.02E-02	3.11E-03	3.11E-02
5	4.44E-02	4.44E-01	1.93E-02	1.93E-01	3.06E-03	3.06E-02	2.49E-03	2.49E-02
10	1.60E-02	1.60E-01	1.64E-02	1.64E-01	1.76E-03	1.76E-02	1.79E-03	1.79E-02
20	7.04E-03	7.04E-02	1.24E-02	1.24E-01	1.35E-03	1.35E-02	1.18E-03	1.18E-02
30	5.11E-03	5.11E-02	9.82E-03	9.82E-02	1.10E-03	1.10E-02	8.81E-04	8.81E-03
40	3.99E-03	3.99E-02	7.99E-03	7.99E-02	9.82E-04	9.82E-03	7.47E-04	7.47E-03
50	3.27E-03	3.27E-02	6.80E-03	6.80E-02	8.99E-04	8.99E-03	6.37E-04	6.37E-03
60	3.16E-03	3.16E-02	5.90E-03	5.90E-02	8.34E-04	8.34E-03	5.68E-04	5.68E-03
70	3.00E-03	3.00E-02	5.09E-03	5.09E-02	8.28E-04	8.28E-03	5.18E-04	5.18E-03
80	2.89E-03	2.89E-02	4.61E-03	4.61E-02	7.69E-04	7.69E-03	4.78E-04	4.78E-03
90	2.87E-03	2.87E-02	4.26E-03	4.26E-02	7.22E-04	7.22E-03	4.49E-04	4.49E-03
100	2.57E-03	2.57E-02	3.85E-03	3.85E-02	6.92E-04	6.92E-03	4.33E-04	4.33E-03
